# Disease severity and efficacy of homologous vaccination among patients infected with SARS‐CoV‐2 Delta or Omicron VOCs, compared to unvaccinated using main biomarkers

**DOI:** 10.1002/jmv.28098

**Published:** 2022-09-09

**Authors:** Ayad M. Ali, Ahmed M. Tofiq, Hassan M. Rostam, Kameran M. Ali, Hassan M. Tawfeeq

**Affiliations:** ^1^ Department of Chemistry University of Garmian Kalar Kurdistan Region Iraq; ^2^ Department of Biology, College of Education Head of International Academic Relations (IRO) University of Garmian Kalar Kurdistan Region Iraq; ^3^ Immunology & Immuno‐Bioengineering Group, Infections, Immunity and Microbes Division, School of Life Sciences, Faculty of Medicine & Health Sciences University of Nottingham Nottingham UK; ^4^ Department of Medicine, College of Medicine University of Garmian Kalar Kurdistan Region Iraq; ^5^ Medical Lab Technology Department, Kalar Technical College Sulaimani Polytechnic University Kalar Kurdistan Region Iraq

**Keywords:** AstraZeneca, Delta variant, homologous vaccination, Omicron variant, Pfizer, SARS‐CoV‐2

## Abstract

From March 2021, various countries including Iraq issued prompted recommendations for increased COVID‐19 vaccine protection in individuals especially those at risk of catching the virus (i.e., lifestyle, health sector workers, and chronic diseases). It is critically important to understand the impact of COVID‐19 vaccinations with the most commonly used vaccines (Pfizer and AstraZeneca) among populations either on the severity of the disease or the transmissibility of SARS‐CoV‐2 variants of concern (VOCs) and in sequential waves. This study was conducted to establish the clinical severity of COVID‐19 caused by Delta and Omicron SARS‐CoV‐2 variants among patients who either attended or were admitted to hospitals and to compare the effectiveness of Pfizer and AstraZeneca COVID‐19 vaccines (single or double doses) at least to prevent hospitalizations if not eradicating the pandemic. A case–control study was done of 570 hospitalized patients; including 328 COVID‐19 confirmed patients (166 males, 160 females) who received homologous vaccinations and 242 unvaccinated patients (128 males, 114 females) during the studied waves. The study showed that unvaccinated COVID‐19 patients in both waves had expressed significantly a higher number and longer periods of symptoms than vaccinated ones. Additionally, there was no significant effect of vaccine types, Pfizer and AstraZeneca or vaccine shot numbers on the PCR‐Ct in the last (Omicron) wave of the pandemic. However, in the previous (Delta) wave of the pandemic, fully vaccinated (double doses) COVID‐19 patients had higher PCR‐Ct values. Whether among vaccinated or unvaccinated patients, lower CRP levels recorded during the Omicron wave than that of the Delta wave, and regardless of the vaccine type or shot numbers, there were no significant differences between the two waves. Lower WBCs were observed in patients (vaccinated and unvaccinated) infected with the Delta variant in comparison to those infected with the Omicron variant and without any remarkable effect of the vaccine type or shot numbers. This is the first molecular and investigational study of the Delta variant and circulated Omicron in Iraq, regarding the severity of these two waves of SARS‐CoV‐2 pandemic and the efficacy of homologous vaccination, indicating the insufficiency of two doses and the demand for booster dose(s) as the most effective way of keeping on the safe‐side against SARS‐CoV‐2.

## 1 INTRODUCTION

In the mid‐November 2019, the Chinese press issued a warning that Wuhan city was attacked by a new, contagious, and life‐threatening viral disease, later named Coronavirus disease 2019 (COVID‐19) caused by severe acute respiratory syndrome coronavirus 2 (SARS‐CoV‐2). The outbreak like wildfire was disseminated over the world in a short span of time, compelling the World Health Organisation (WHO) to declare it a global pandemic in early 2020.^1^, ^2^ The disease has continued to exhibit devastating consequences resulting in more than 5.8 million deaths worldwide in Feb. 2022^3^ emerging as the most global health crisis since the era of the influenza pandemic of 1918.^4^ In South Africa, and in a hospital system, when researchers conducted comparison research for the health outcomes throughout four sequential waves of COVID‐19, they defined every wave as the duration of time when positivity proportions outreached 26%.^5^

It appears that Omicron is with a much higher average of asymptomatic cases over other variants of concern (VOC), which might interpret its widespread, quick prevalence, even among inhabitants with high previous percentages of SARS‐CoV‐2 infection.^6^ Concerning signs and symptoms of infection with Omicron and the difference to the infection with prior SARS‐CoV‐2 variants, it has been reported that besides a headache, runny nose, fatigue (both mild and severe), sore throat, sneezing, as the five most known symptoms for Omicron, and the newly announced anorexia and brain haze (more popular in individuals who were completely vaccinated and boosted), extra reports indicated night sweats as well.^7^

It is obvious that regardless of the difference between asymptomatic and symptomatic COVID‐19, a meaningful part of asymptomatic natural attacks stimulates humoral immune response providing the capability to resist reinfection.^8^

Substantial progress in clinical research has led to a better understanding of the management of SARS‐CoV‐2, limiting the spread of the virus and its variants has become an issue of increasing concern, as SARS‐CoV‐2 continues to wreak havoc across the world, with many countries enduring a four‐wave of outbreaks, mainly due to the emergence of mutant variants of the virus,^9^, ^10^ that may have different characteristics than its ancestral strains. Among SARS‐CoV‐2 variants, only a few are considered VOCs by the WHO, given their impact on global public health as of December 11, 2021, five (alpha, beta, gamma, delta, and Omicron) strains of SARS‐CoV‐2 have been identified by WHO as VOCs since the beginning of the pandemic.^11^, ^12^, ^13^

From mid‐2021 to march, 2022, two waves of COVID‐19 hit Iraq, including the Kurdistan Region, considered the third (W3) and fourth (W4) waves of the pandemic. The first case of the SARS‐CoV‐2 Delta (B.1.617.2) variant led to the third wave was recorded in July 2021.^14^ Molecular biology techniques used for the identification of SARS‐CoV‐2 isolates from Sulaimani province in Iraq's Kurdistan Region, revealed two distinct variants. The former variant turns out to be from the next clade 21 J (Delta), GISAID clade O, and VOC Delta (B.1.617.2.), while the other belongs to the next clade 21 (Omega), GISAID clade GRA, and VOC Omicron (B.1.1.529+BA.1).^15^

After 2 years of the COVID‐19 pandemic, health systems worldwide have still not achieved control of the disease. SARS‐CoV‐2 is highly transmissible with a potential secondary attack rate of more than 17%.^16^ This rate of transmission has been reported to be even higher in circulating VOC such as the B.1.1.7 than in pre‐existing variants.^17^ Since its emergence in December 2019, the SARS‐CoV‐2 virus has infected more than 535 million people and led to at least 6 million deaths globally.^18^ In addition to the high disease burden, the virus has brought an unprecedented downpour of social and economic setbacks, the course of which cannot start to be reversed until herd immunity, natural or artificial, is achieved. While six vaccines are already licensed, we are still far from herd immunity, given that vaccines need to be produced at scale, priced affordably, and allocated globally to be widely deployed.^19^, ^20^ Expeditious identification of clinical hazard factors, underlying health conditions or comorbidities, and serious consequences which can anticipate advancement in the vicinity of the severe form of the illness (COVID‐19) is preeminent for on‐time intervention to hinder fatal outcomes.^21^, ^22^ Additionally, in consideration of emerging variants and reports of recurrent SARS‐CoV‐2 infection, the global battle against the virus is far from being over.^23^, ^24^

COVID19 vaccination is recognized to be adequate to eradicate the pandemic burden. The population's willingness is to control vaccination programs related to their vaccine acceptance. Limited studies are published highlighting this acceptance.^25^ In the present study, we aimed to assess the efficacy of homologous COVID‐19 vaccination on the severity of the disease and the transmissibility of SARS‐CoV‐2 among patients infected with SARS‐CoV‐2 Delta or Omicron VOCs.

## 2 MATERIALS AND METHODS

### 2.1 Study design and patients

This study was designed regarding the COVID‐19 pandemic waves (W3 and W4 in Iraq) with clinical and biological parameters for patients. The first study included 328 COVID‐19 confirmed patients (166 males, 160 females) who were taken vaccination (single or double dose messenger RNA [mRNA] vaccination). The second study included 242 patients (128 males, 114 females) that did not vaccinate. The study was conducted at Qalla Hospital for COVID‐19, Kalar, Kurdistan Region, Iraq.

### 2.2 Biological parameters

In this study, some biological markers examined included PCR Ct‐values, CBC, CRP, and signs and symptoms between the two waves for patients. The CBC was primarily performed using a Medonic M‐Series hematology analyzer (Medonic M32; Boule Medical AB). CRP test was conducted on an automated multiparametric analyzer Cobas C111 4 (Roche Diagnostics).^26^, ^27^

Clinical classification of Severity Case Category of patients was based primarily on the Diagnosis and Treatment Protocol for Novel Coronavirus Pneumonia (Trial Version 7) (developed by the National Health Committee of the People's Republic of China) National Health Commission of the People's Republic of China home page. Available from: http://www.nhc.gov.cn. Classifications are characterized as follows: (1) mild type: only mild clinical symptoms with no sign of pneumonia in imaging features; (2) moderate type: complicated with fever, respiratory symptoms, and imaging features of pneumonia; (3) severe type: complicated with any of the following: respiratory distress; respiratory rate ≥ 30 beats/min, mean oxygen saturation ≤ 93% at rest, or ratio of the partial pressure of arterial oxygen to the fraction of inspired oxygen (PaO_2_:FiO_2_) ≤300 mmHg (1 mmHg = 0.133 kPa).

### 2.3 Reverse‐transcription polymerase chain reaction (RT‐PCR)

For detection of SARS‐COV2, real‐time PCR was carried out using the extracted viral RNA template from the nasopharyngeal swab sample, the procedure was carried out in previous work.^28^

### 2.4 Ethics declarations

All methods were carried out in accordance with relevant guidelines and regulations. We confirm that all experimental protocols were approved by the Ethics Licensing Committee of the Kalar Technical Institute at the Sulaimani Polytechnic University (No. 03 on 02/03/2021). In addition, informed consent was obtained from all participants or from a parent or legal guardian if participants were under the age of 18.

### 2.5 Statistical analysis

A two‐way analysis of variance test by GraphPad Prism 9.3 was used to observe the statistical differences between the control and test group for all parameters. In addition, Analyse‐it software in Microsoft Excel 2020 was used to perform Principal Component Analysis (PCA)‐Biplot to see any correlation between patient comorbidities and treatments with COVID‐19 patient groups.

## 3 RESULTS

**Figure 1:**
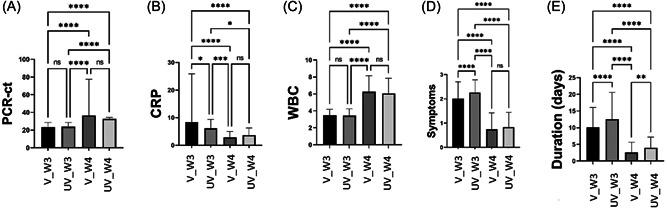
Comparison between vaccinated (V) and unvaccinated (UV) COVID‐19 patients during the 3rd wave (W3) and 4th wave (W4) in patients PCR‐Ct value, polymerase chain reaction–cycle threshold (A), CRP, C‐reactive protein (B), WBC, white blood cell (C), symptom numbers (D) and symptom duration (E).

In the current study, there were no significant differences in PCR‐Ct value between vaccinated and unvaccinated COVID‐19 patients in the 3rd and 4th waves of the pandemic. However, the PCR‐Ct value in the 4th wave of the pandemic (in vaccinated and unvaccinated COVID‐19 patients) was significantly (*pv* < 0.0001) higher than Vaccinated COVID‐19 patients of the previous wave of the disease (Figure 1A).

CRP in vaccinated COVID‐19 patients (8.43 ± 17.48) was significantly (*pv* = 0.01) higher than in unvaccinated COVID‐19 patients (6.14 ± 3.27) in the 3rd wave of the pandemic, but there were no significant differences of CRP between vaccinated and unvaccinated COVID‐19 patients in a later wave of the disease. Furthermore, CRP in the vaccinated COVID‐19 patients in the third wave was significantly higher than in vaccinated COVID‐19 patients (2.83 ± 2.21), *pv* ≤ 0.0001) and unvaccinated COVID‐19 patients (3.67 ± 2.60, *pv* < 0.0001) in 4th wave of the pandemic. Also, CRP in unvaccinated COVID‐19 patients in the 3rd wave (6.14 ± 3.27) was significantly higher than CRP in vaccinated (2.83 ± 2.21, *pv* = 0.0005) and unvaccinated COVID‐19 patients (3.67 ± 2.60, *pv* = 0.01) during the 4th wave of the pandemic (Figure 1B).

WBC count in vaccinated and unvaccinated COVID‐19 patients was similar in both waves of the pandemic, even though the WBC count in the third wave (vaccinated, 3.48 ± 0.73 and unvaccinated, 3.44 ± 0.78) was significantly (*pv* < 0.0001) lower than the 4th wave for the vaccinated (6.26 ± 1.90) and unvaccinated (6.06 ± 1.79) COVID‐19 patients (Figure 1C).

Unvaccinated COVID‐19 patients in the 3rd waves had expressed a significantly (*pv* < 0.0001) higher symptom (2.26 ± 0.53) than vaccinated COVID‐19 patients (2.01 ± 0.69); 4th wave, mean ± SD). Interestingly, there were not any significant differences between vaccinated and unvaccinated patients in the 4th wave of the pandemic. Generally, the symptoms in the 4th wave were significantly less than in the 4th wave (Figure 1D).

Symptomatic period of vaccinated COVID‐19 patients (3rd wave, 10.13 ± 5.96; 4th wave (2.57 ± 3.04) was significantly shorter in comparison with unvaccinated COVID‐19 patients (3rd wave, 12.53 ± 8.11, *pv* < 0.0001; 4th wave, 3.99 ± 3.19, *pv* = 0.001) in each wave. Interestingly, the symptomatic period in the 4th wave was significantly (*pv* < 0.0001) shorter than the 3rd wave of pandemics in vaccinated and in unvaccinated patients (Figure 1E).

**Figure 2:**
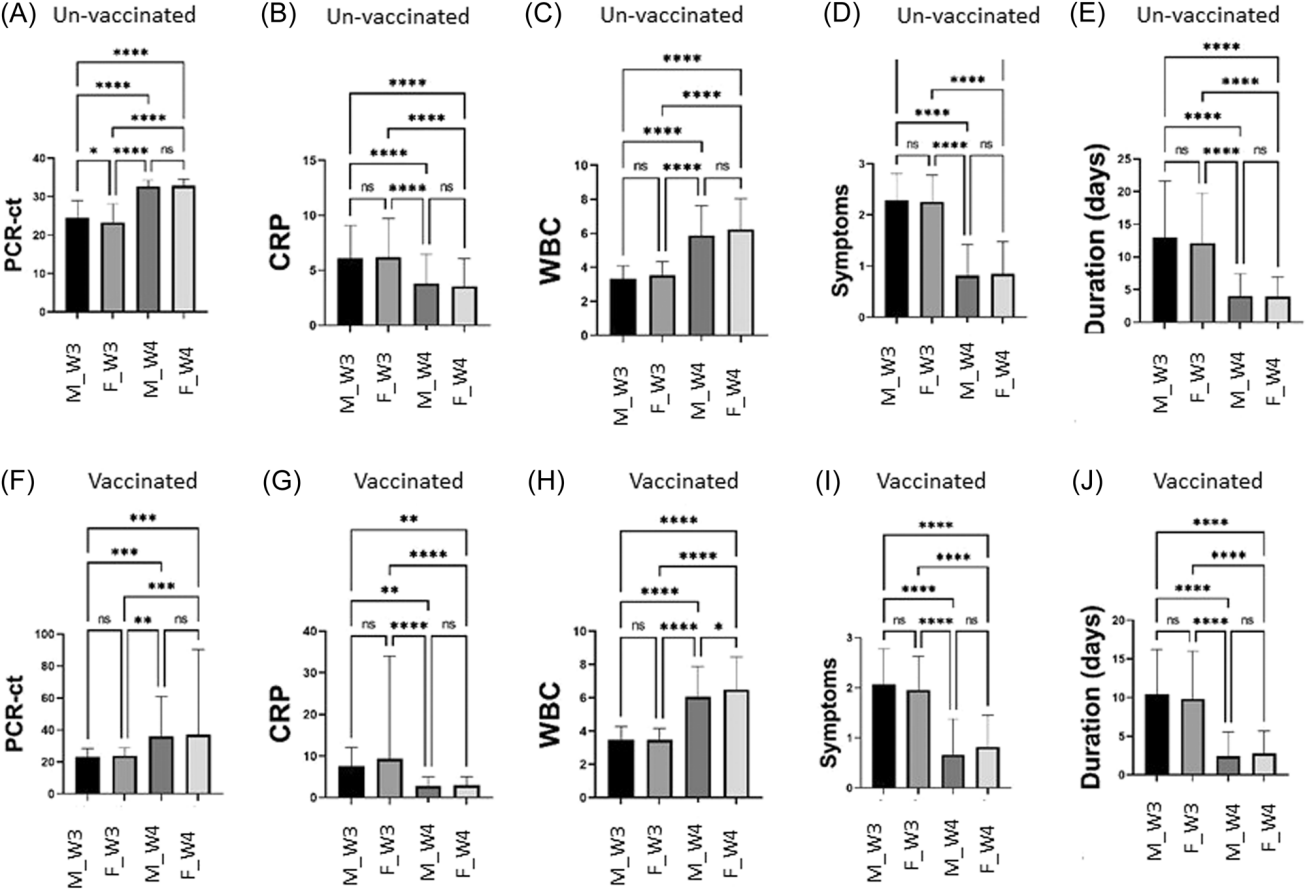
Comparison between both gender, male (M) and female (F) during the 3rd wave (W3) and 4th wave (W4) in unvaccinated (A–E) and vaccinated (F–J) COVID‐19 patients in their PCR‐Ct value, polymerase chain reaction–cycle threshold (A, F), CRP, C‐reactive protein (B, G), WBC, white blood cell (C, H), symptom numbers (D, I), and symptom duration (E, J).

In the current study, there were not any significant differences between unvaccinated COVID‐19 males and females in both waves for all studied parameters, except for the PCR‐Ct value in the 3rd wave where males had a significant (*pv* = 0.01) higher PCR‐Ct value (24.5 ± 4.38) than female (23.31 ± 4.79) (Figure 2).

Vaccinated females in 4th wave of the COVID‐19 pandemic experienced a significant increase (*pv* = 0.01) in WBC number (6.48 ± 1.94) when compared with vaccinated males (6.05 ± 1.83) in the same wave, otherwise, there was no a statistical difference between both gender in both waves for all other parameters (Figure 2).

The current study shows no effect of differences in vaccine types (Pfizer and AstraZeneca) or vaccine shot numbers (single and double) on the PCR‐Ct value in the 4th wave of the pandemic. However, in the 3rd wave of the pandemic, double vaccinated (AstraZeneca, 30.0 ± 2.27; Pfizer, 25.67 ± 4.51) COVID‐19 patients had a higher PCR‐Ct value than single vaccinated (AstraZeneca, 24.15 ± 5.04; Pfizer, 22.38 ± 5.03) patients in the same wave.

During the 3rd wave of the pandemic, Pfizer single vaccinated COVID‐19 patients had a higher CRP value (9.34 ± 21.48) than Pfizer double vaccinated COVID‐19 patients (7.52 ± 4.37, *pv* = 0.01) and AstraZeneca single vaccinated COVID‐19 patients (5.52 ± 3.72, *pv* < 0.0001).

**Figure 3:**
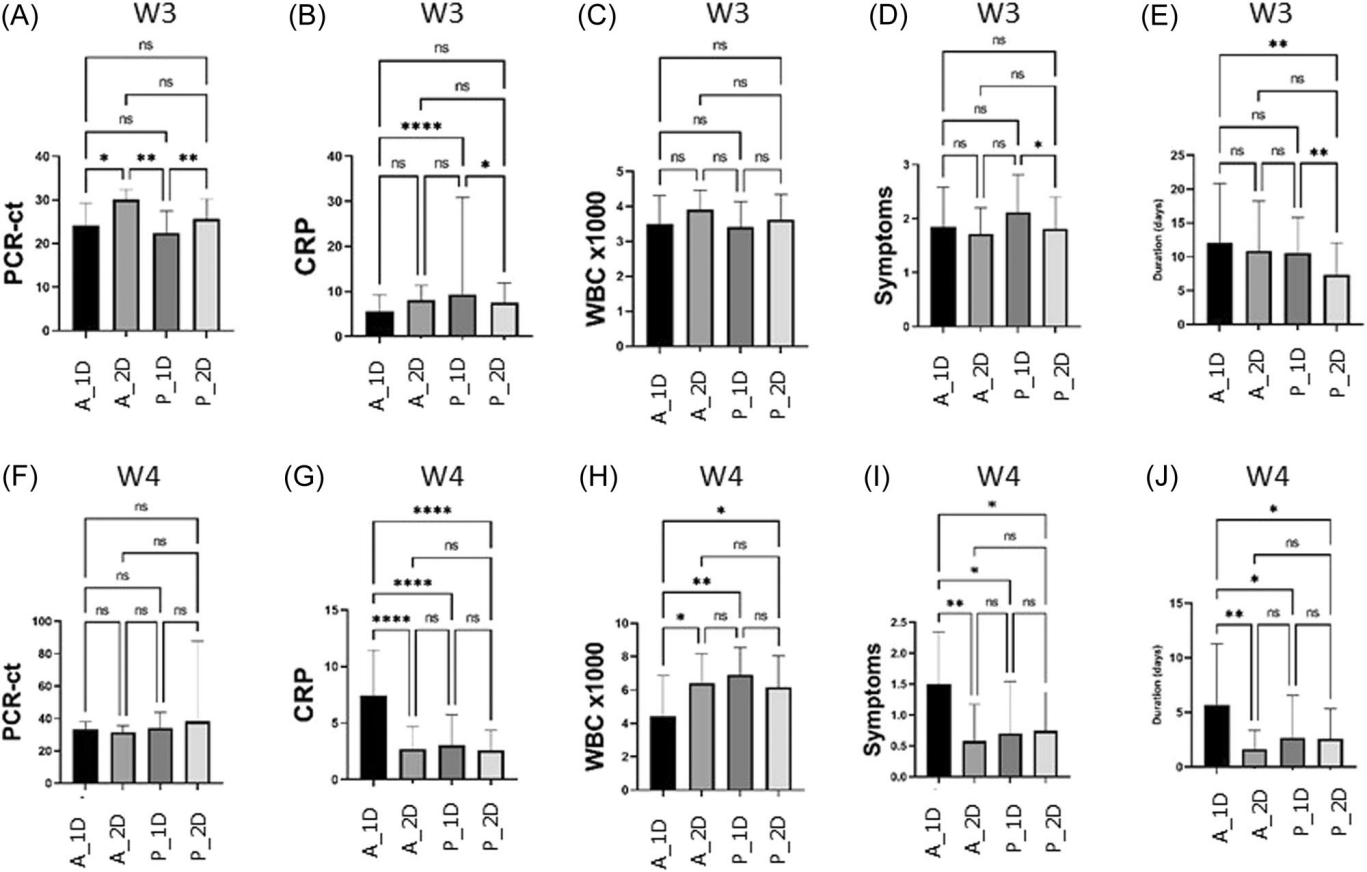
Comparison between both COVID‐19 vaccinated (AstraZeneca [A] and Pfizer [P] in COVID‐19 patients who received a single dose [1D] and double dose [2D] during) the 3rd wave (W3) (A–E) and 4th wave (W4) (F–J) in their PCR‐Ct, polymerase chain reaction–cycle threshold (A, F), CRP, C‐reactive protein (B, G), WBC, white blood cell (C, H), symptom numbers (D, I), and symptom duration (E, J).

In the 4th wave of the COVID‐19 pandemic, AstraZeneca single vaccinated COVID‐19 patients had a higher CRP (7.45 ± 3.99) than AstraZeneca double vaccinated COVID‐19 patients (2.68 ± 2.01, *pv* < 0.0001), Pfizer single (2.68 ± 2.01, *pv* < 0.0001) and double (2.59 ± 1.75, *pv* < 0.0001) vaccinated COVID‐19 patients. In the 4th wave of COVID‐19 patients who had a single jab of AstraZeneca COVID‐19 vaccine showed lower WBC numbers (4.42 ± 2.42) than AstraZeneca double vaccinated COVID‐19 patients (6.41 ± 1.78, *pv* = 0.01) Pfizer single vaccinated (6.90 ± 1.63, *pv* = 0.001) And Pfizer double vaccinated (6.16 ± 1.89, *pv* = 0.01). However, there were not any significant differences between single or double‐vaccinated COVID‐19 patients for/between both vaccinated (Figure 3).

Symptomatic period duration and the number of the COVID‐19‐related symptoms in the 4th wave of COVID‐19 pandemic was significantly decreased to half in Pfizer (single and double) and AstraZeneca double vaccinated COVID‐19 patients when compared to AstraZeneca single vaccinated COVID‐19 patients.

Whoever, in the 3rd wave of the pandemic, was no any significant effect of types of vaccine or number of jabs on the number of COVID‐19 related symptoms among vaccinated COVID‐19 patients, except for Pfizer single vaccinated COVID‐19 who had higher number of related symptoms than Pfizer double vaccinated COVID‐19 patients, who had a lower symptomatic period than Pfizer and AstraZeneca single vaccinated COVID‐19 patients (Figure 3).

**Figure 4:**
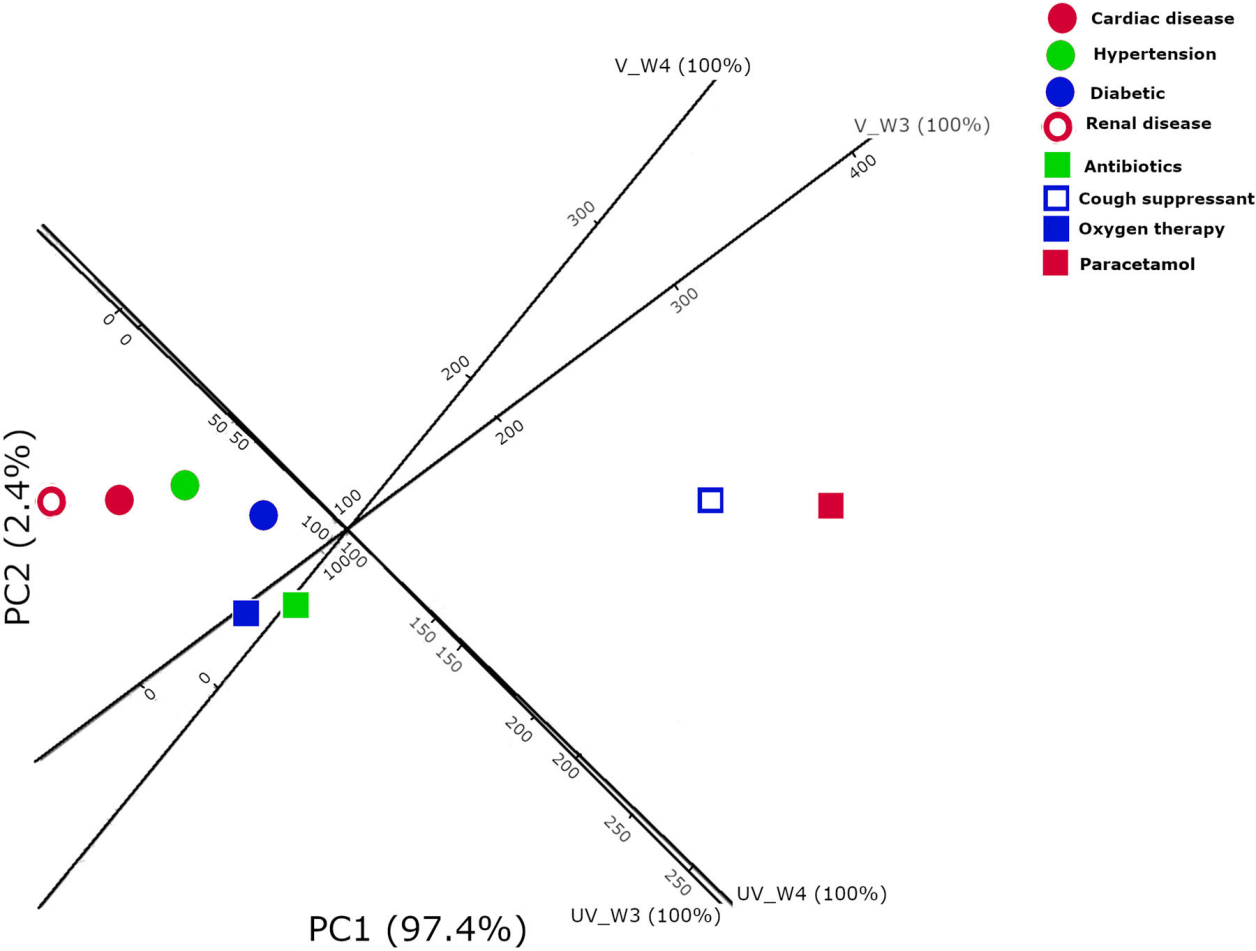
Principal component analysis in COVID‐19 patients groups (V_W3) vaccinated 3rd wave, (V_W4) vaccinated 4th wave, (UV_W3) unvaccinated 3rd wave, and (UV_4W) unvaccinated 4th wave for possible confounding factors (comorbidities; cardiac disease, diabetics, hypertension and renal disease) and treatments (antibiotics, cough suppressant, paracetamol, and oxygen therapy) used.

PCA shows that no significant correlation between COVID‐19 patient comorbidities including cardiac disease, diabetics, hypertension, and renal disease in vaccinated or unvaccinated patients in both waves (3rd and 4th) of the pandemic. Also, there was no significant correlation between COVID‐19 treatment with a patient group in comparison with other study groups Figure 4.

## 4 DISCUSSION

Since the COVID‐19 pandemic swept the world, the global regimen has changed; benefiting from non‐pharmaceutical interventions (NIPs), for example, the use of facemasks, keeping physical distances, community stay‐at‐home measures, regular hand washing and sanitizing, isolation, and quarantine, on one side, and significant efforts to build herd‐immunity threshold, as public‐health policymakers to eradicate and stop spreading of infectious disease (i.e., the pandemic) through vaccination on another side.^29^ Although successive COVID‐19 waves have spread throughout the world, out of all the VOCs (Alpha, Beta, Gamma, Delta, and Omicron),^30^ SARS‐CoV‐2 Omicron (B.1.1.529) observed the largest number of mutations and are able to bypass the immune response to the highest extent. The World Health Organization rapidly designated this recently emerged variant as a VOC which has been first identified in Botswana (South Africa), in late November, 2021.^31^, ^32^ Rasmussen illustrated that vaccination against COVID‐19 was primarily proposed as the sole viable avenue to gain herd immunity for this respiratory viral infection instead of natural infection, as long as herd immunity is demanding for everlasting control of the pandemic.^33^ Initial distribution of the infection, the features of vaccines, the scope of the herd immunity, and the power of NIPs, are the main factors that the control of the pandemic depends on. In this study, we tested both the severity and breakthrough infections of SARS‐CoV‐2 VOC in the third and fourth waves (Delta and Omicron) of the pandemic in 242 unvaccinated patients and 328 vaccinated patients. Also, to show the extent of vaccine effectiveness (VE) or efficacy in individuals vaccinated with either a single or two doses (fully vaccinated) of either the mRNA (Pfizer, Moderna) or Ad vector (adenovirus (Ad)‐vectored) of AstraZeneca vaccines to provoke the quick production of neutralizing antibodies (NAbs).

Regardless of the pandemic waves, there were no significant differences between vaccinated and unvaccinated SARS‐CoV‐2 patients concerning viral burden (Ct‐values). However, vaccinated and unvaccinated patients in W4 had lower viral burden (higher Ct values) than vaccinated patients of the previous wave. Researchers showed that there are two discrete types of SARS‐CoV‐2 infections during 2021, a group with a low viral burden that dominated earlier in 2021, and a group with a high viral burden (lower Ct values), which increased in recurrence with Delta.^34^, ^35^, ^36^ Also, it has been pointed out that in the Delta‐dominant period, VE reduced, and there was a considerable shift in peak viral load in individuals infected in spite of vaccination with two doses of either BNT162b2 or ChAdOx1, with almost identical mean Ct values to individuals infected without vaccination.^37^ Our current findings expand upon prior work,^38^ that in infected patients, vaccinated and unvaccinated, the peak viral load appears to be similar, with the risk of potential implications for ahead transmission, due to the strong connection between peak Ct values and infectivity. To make a comparison between omicron and delta infections, a Scottish study determined November 23 to December 19, 2021, as the date was the first case of Omicron reported by the national surveillance data.^39^ It is agreed that the overall number of infections is presumed to decrease significantly as the number of individuals acquiring natural immunity increases, which in turn must minimize the rate of transmission. However, an exception of genetic modifications in the circulating variants including 15 and 11 mutations in the receptor‐binding domain (RBD) and the N‐terminal domain (NTD), respectively, may increase the virus transmissibility, the chances of reinfection, and fractional resistance to the ongoing vaccines, but inherently with less severity.^40^, ^41^, ^42^, ^43^, ^44^

Considering biomarkers as explanatory variables, such as SARS‐CoV‐2 patients' CRP levels and WBCs were measured in Delta and Omicron of the current study. It appears that SARS‐CoV‐2 induces an “imbalance host immune response”^45^ in respect of the production of various cytokines like IL‐1, IL‐2, IL‐3, IL‐4, IL‐6, IL‐10, IL‐17, and interferon INF‐I, INF‐II, INF‐III, and TNF‐α. These cytokines can lift CRP levels which are pointers of inflammation and are more likely to influence viral elements.^46^ Our data indicate elevated levels of CRP during Delta in vaccinated over unvaccinated patients in the same wave and even over the levels of CRP in vaccinated and unvaccinated patients of the next wave (Omicron) as well. This is consistent with the last report presented by the UK Health Security Agency, that the risk among the Omicron cohort to emergency care or hospitalization was generally half of that for the Delta cohort (hazard ratio: 0.53, 95% confidence interval [Cl]: 0.50–0.57).^47^ Also, the risk of hospitalization from the emergency care units in the Omicron cohort was roughly one‐third of that for the Delta cohort (hazard ratio: 0.33, 95% Cl: 0.30–0.37). Therefore, levels of CRP are thought to be a preliminary benchmark of pneumonia and the severity of COVID‐19.^47^, ^48^ We observed similar WBCs in vaccinated and unvaccinated patients in both waves of the pandemic, however, the Delta cohort (vaccinated and unvaccinated) showed lower WBCs than the next wave. Even though the prevalence of the Omicron variant in South Africa was very quick, in a populace wherein 60%–80% already displays serological proof of previous infection or vaccination, proposing that this variant is capable to bypass immunity gained naturally or induced by vaccination.^49^ Our data is in line with that of Ali et al.^27^ that the levels of CRP and WBCs were increased between hospitalized COVID‐19 patients, and more accurately throughout the second week of hospital admission, as a result of the initiation of the immune response. Moreover, elevated levels of CRP and WBCs in human blood could explain to great extent the severity of COVID‐19 infection.^50^

It is evident from the results associated with symptoms and the period (duration) of the symptoms in vaccinated and unvaccinated SARS‐CoV‐2 patients, and for both of the two sequential waves, that unvaccinated patients had expressed higher symptoms and for a longer period than vaccinated ones. Moreover, vaccinated and unvaccinated patients in W3, and in comparison to those in W4, had experienced higher and longer‐lasting symptoms.^51^ This approach is in contradiction to that of Pouwels et al.,^52^ that during the Delta‐dominant period, significantly more identical percentages of announced symptoms, pushed by Ct (higher viral burden), were detected both in infected vaccinated and unvaccinated individuals.

In comparison to fully vaccinated individuals, the transmission for unvaccinated individuals was higher and diminished in individuals with booster vaccinations. In Omicron and Delta households, the secondary attack rate (SAR) for unvaccinated individuals was 19% and 28%, sequentially, while the SAR in individuals with full vaccination was 32% and 19%. However, the SAR in individuals with booster vaccination was 25% and 11% in Omicron and Delta, respectively. This data is in line with our data reported earlier. As stated in these findings, it can be concluded that the rapid dissemination of Omicron is attributed primarily to the immune escape instead of the increase in the essential transmissibility of the virus.^53^

Apparently, there is no reasonable difference among unvaccinated male and female individuals infected with SARS‐CoV‐2 during Delta and Omicron waves regarding the studied parameters (PCR‐Ct value), CRP, WBCs, symptoms, and duration of symptoms, except those infected males in Delta experienced higher PCR‐Ct value over females. Similarly, there was no statistical difference when infected but vaccinated male and female individuals were compared using the same parameters. However, in W4 vaccinated females showed higher WBCs than male ones.

Regardless of age, sex, and body mass index, disease symptoms persist at an early SARS‐CoV‐2 infection stage was remarkably positively corresponded with stronger and extra sustained ant‐RBD‐IgG quantities.^40^ Samples obtained from formerly fully vaccinated and unvaccinated people who were infected during the period of the Omicron wave in South Africa were analyzed for the evaluation of whether neutralizing immunity drawn out by Omicron strengthened neutralizing immunity of the Delta variant.^54^ Apparently, the neutralization power of the live virus of Omicron boosted 14‐fold from registration to an average of 14 days, in addition to strengthening Delta virus neutralization, which boosted 4.4‐fold. Thus, the increase in the neutralization of Delta variant in individuals who recovered from infection with Omicron might end in decreasing the potential of Delta VOC to reinfect those individuals. This prediction supports our results that the data for all the parameters during Omicron are less than those in the previous wave, Delta.

A qualitative and quantitative analysis to determine the effectiveness of the vaccination; types (Pfizer and AstraZeneca) and doses (single or double [fully]) of the vaccines were applied. There was no effect of either the differences of the vaccine type or vaccine shot numbers on the PCR‐Ct value during the Omicron attack, whereas fully vaccinated (Pf or AZ) patients showed higher Ct value than single vaccinated ones during the Delta attack. This verifies that full vaccination had a meaningful impact on a patient's immune system by reducing viral burden in Delta compared to Omicron. However, during the Omicron period, the Ct value in fully vaccinated individuals with Pf slightly exceeds those who received Pf (single dose), and AZ (single and double doses). This might indicate to some extent that Pf tends to be a better positive immunogenic effect than AZ. The current result is validated by the presence of evidence that vaccination drastically reduces the viral burden and symptomatic or asymptomatic attacks in vaccinated people, which could be translated into a decreased transmission, despite the fact that the vaccine efficacy differs by vaccine product and intention group.^40^ Monitoring periods for vaccinated individuals are not so far adequately quite a long time to sketch conclusions on the period of protection towards infection long‐lasting. Following vaccination, antibody titers require 3−4 weeks to reach a peak in vaccinated individuals.

In W3, Patients receiving a single dose of Pf had higher CRP levels than those receiving either two doses of Pf or a single dose of AZ, which is unexpected but may have been due to other microbial infections in addition to COVID‐19. However, it should be noticed that the immune system of patients with a single dose of vaccination had not been induced adequately to have strong protection against SARS‐CoV‐2. On the hand, COVID‐19 patients during W4 with a single dose of AZ marked higher CRP levels than those fully vaccinated with AZ, and single and double vaccinated with Pf, which was predictable and meaningful; our data were comparable with previous work of Özüdoğru et al.^55^

Concerning WBCs, no significant differences between single or double vaccinated SARS‐CoV‐2 patients for/between both vaccine types were identified in each of Delta and Omicron. Despite this, in W4 patients receiving a single dose of AZ showed fewer WBCs than those double vaccinated with AZ, and single and double vaccinated with Pf. Out of 226 patients infected with SARS‐Cov‐2 Delta VOC (B.1.167.2), consisting of 77 unvaccinated children (aged under 12 years) and 149 individuals aged 12 years and over, mostly vaccinated, recorded with higher peripheral blood lymphocyte counts, and higher normal CRP rate.^40^ These data support our findings.

It has been found that vaccine effectiveness after a single dose of either Pfizer or AZ was notably lesser among individuals with the Delta variant (30.7%). However, only the slightest differences in vaccine effectiveness were identified with the Delta variant after two doses of either vaccine.^56^ There is a concern that the number of mutations on the S protein of the Omicron variant, may confer the capability of this variant to weaken vaccine effectiveness, and theory suggests that the potency of Abs produced through vaccination will be diminished.^56^ Recent scientific knowledge proves that Omicron markedly escapes vaccine‐induced immune response after an initial vaccination regimen with Pfizer and AZ vaccines and displays higher infectivity, raising the prospects for increased transmissibility.^31^, ^57^ Moreover, in vitro studies using sera gathered from individuals fully vaccinated with either BioNTech, Pfizer vaccine (BNT162b2), or Moderna mRNA‐1273 vaccine, demonstrated 11.4‐ and 20‐fold reduction in antibody neutralization capacity towards Omicron.^58^ However, using sera obtained from individuals vaccinated with the ChAdOx1 nCoV‐19 vaccine, there wasn't any observable neutralization efficacy. These data suggest an elevated hazard of reinfection as a result of the limitation in antibody‐mediated neutralization against this SARS‐CoV‐2 variant.

COVID‐19 patients with a history of hypertension, diabetes, cardiovascular disease, obesity, chronic lung disease, chronic kidney diseases, cancer, and elderly patients in long‐term care units are at high risk of contracting the virus and have a worse disease prognosis, even the risk of death among these groups.^21^, ^22^, ^59^ Moreover, each antibiotic, antiviral drugs, and glucocorticoids were with a significantly higher incidence of treatment in COVID‐19 patients.^20^ To eradicate the possibility of the effect of morbidity and treatment on our study groups, a PCA of possible confounding factors including comorbidities and treatments in correlation with studied groups has been performed. Our data has not revealed any significant correlations between underlying comorbidities (single and multiple) or specific treatment with any studied group in comparison with other groups; vaccinated, unvaccinated, Delta, and Omicron.

To conclude, the emergence of Omicron illuminates the challenges facing all types of vaccines, as their designs were based on the genomic sequence of the wild‐type strain of the virus from Wuhan.^31^ The risk of hospitalization among individuals infected with Omicron vaccinated with double doses was quite similar to among individuals infected with Delta variant, emphasizing the significance of booster doses launch.^60^ Moreover, the spike of the Omicron variant increases the tendency for reverse zoonosis, and is with high potential compared to other variants to initiate SARS‐CoV‐2 animal reservoir,^40^ and reduces the chances of SARS‐CoV‐2 ever being eradicated. Additionally, in consideration of emerging variants of recurrent SARS‐CoV‐2 infections, the global battle against the virus is far from being over.^23^ Future lines of research must focus on the efficacy of full vaccination, booster (third and fourth) doses with heterologous vaccines, and perform genomic sequencing in all SARS‐CoV‐2 cases. We highly recommend human society, in particular, the health sector, consider the outcomes of this study, and the risk of comorbid conditions associated with the pandemic.

## AUTHOR CONTRIBUTIONS

All authors conceived of the idea and planned the experiments; Ayad M. Ali performed the lab work with data collection. Hassan M. Rostam has performed data analysis. All other authors equally participated in writing, reviewing, and editing the manuscript.

## CONFLICT OF INTEREST

The authors declare no conflict of interest.

## Data Availability

The data that support the findings of this study are available from the corresponding author upon reasonable request.
